# Adolescent exposure to food and beverage marketing on social media by gender: a pilot study

**DOI:** 10.1017/S1368980022002312

**Published:** 2022-11-02

**Authors:** Ashley Amson, Elise Pauzé, Lauren Remedios, Meghan Pritchard, Monique Potvin Kent

**Affiliations:** 1 University of Ottawa, Interdisciplinary School of Health Sciences, Ottawa, ON, Canada; 2 University of Ottawa, School of Epidemiology and Public Health, 600 Peter Morand, Room 301J, Ottawa, ON K1N 7K4, Canada

**Keywords:** Adolescents, Gender, Food marketing, Marketing techniques, Social media

## Abstract

**Objective:**

The objective of this research was to determine if, based on gender, adolescents were exposed to different marketing techniques that promoted food and beverages over social media.

**Design:**

A secondary analysis of adolescent boy (*n* 26) and girl (*n* 36) exposures (*n* 139) to food and beverage marketing was conducted. Mann–Whitney *U* and Fisher’s exact tests were conducted to compare the number, healthfulness and the marketing techniques of exposures viewed by boys and girls.

**Setting:**

Ottawa, Ontario, Canada.

**Participants:**

Sixty-two adolescents aged 12–16 years.

**Results:**

Boys and girls were exposed to similar volumes of food marketing instances (median = 2 for both boys and girls, Mann–Whitney *U* = 237, *P* = 0·51) per 10-min period of social media use. More girls viewed products that were excessive in total fat compared to boys (67 % *v*. 35 %, *P* = 0·02). Boys were more likely to view instances of food marketing featuring a male as the dominant user (50 % *v*. 22 %, *P* = 0·03), appeals to achievement (42 % *v*. 17 %, *P* = 0·04), an influencer (42 % *v*. 14 %, *P* = 0·02) and appeals to athleticism (35 % *v*. 11 %, *P* = 0·03), whereas girls were more likely to view instances of food marketing featuring quizzes, surveys or polls (25 % *v*. 0 %, *P* = 0·01).

**Conclusions:**

Food and beverage companies utilise marketing techniques that differ based on gender. More research examining the relationship between digital food and beverage marketing and gender is required to inform the development of gender-sensitive policies aimed at protecting adolescents from unhealthy food marketing.

Obesity is a pervasive public health concern, increasing an individual’s risk of CVD, type II diabetes and poor self-esteem^(1)^. In 2019, roughly 25 % of all Canadian adolescents aged 12–17 years were classified as being overweight or having obesity, with higher rates of obesity in males (28·5 %) compared with females (20·2 %) based on self-reported anthropometric data^(2)^. In the last few decades, the diets of Canadian adolescents have shifted to include more processed and packaged foods that are high in sugar, salt and/or saturated fat^(3)^. In 2015, 64 % of Canadian adolescents exceeded the recommended daily intake of added sugar, with males aged 6 to 17 years consuming more than their female counterparts (84 g *v*. 76 g)^(4)^. Similarly, males aged 14 to 18 years consumed more sodium per d than females (3320 mg *v*. 2350 mg)^(5)^. These dietary habits can be attributed to several environmental factors including the digital marketing of cheap, highly processed, palatable, nutrient-poor food viewed on smartphones, tablets and computers^(6)^. Differential exposures to digital marketing, as viewed by male and female adolescents, may be contributing to dietary disparities between these groups.

Digital media operates in a complex, interactive web of sharing and collecting information, providing a unique space for companies to advertise their products as it is low cost, and it can be tailored to specific audiences^(7)^. As ‘digital natives’^(8)^ adolescents are born and raised in a media-driven world, where digital devices are a staple of their daily lives. As of 2014, 85 % of Canadians in grade 11 owned a cellphone^(9)^. In 2017, 20 % of Canadian adolescents spent 5 or more hours a day on social media with most adolescents cycling through one to three social media applications (apps) daily^(9)^. Gender differences exist in social media and online use amongst those in grades 7–11. YouTube is used by a larger share of Canadian boys compared with girls (83 % *v*. 77 %). Conversely, Facebook and Twitter are more popular among girls than boys (77 % *v*. 72 % and 43 % *v*. 24 %, respectively)^(9)^. Further, boys are more likely to play online games (71 % *v*. 47 %), while girls are more likely to use social media to connect with others (45 % *v*. 36 %) and to follow celebrities (26 % *v*. 14 %)^(9)^. Overall, young Canadian girls have a stronger presence on social media sites than their male counterparts^(9)^.

Adolescents are a unique sub-population that have not garnered the same attention as children when it comes to the marketing of unhealthy food or beverages, yet they are similarly impacted^(10)^. Research demonstrates that unhealthy food and beverage marketing targeting adolescents directly affects their food preferences, purchasing habits and short-term food intake^(10)^. Corporations target adolescents as they often have greater flexibility to make their own purchases, have increased agency over their decisions and display greater brand loyalty than other age groups, while being impressionable and impulsive^(11)^. Adolescents are also overlooked when it comes to regulatory policies^(10,12)^, leaving them in a more precarious position than children^(12)^. The combination of these factors amplified by adolescents’ extensive use of social media makes them an ideal target population for food and beverage companies. One area that requires attention is the role gender plays in the digital marketing of food and beverages (henceforth collectively referred to as food) to adolescents.

Gender is the social construction of behaviours, roles and activities that are deemed socially appropriate for men and women, boys and girls, and gender-diverse people^(13)^. Gender is also a social determinant of health that can influence health status and reinforce social and cultural norms^(14)^. These norms can change food and dietary choices, which can lead to the adoption of unfavourable health behaviours such as the excessive consumption of unhealthy foods^(14)^. There is a paucity of research examining how food marketing impacts people based on their gender.

A recent scoping review suggests that there is a relationship between gender and food marketing^(15)^. Some of the key findings from the review indicate that: male and female children and adolescents respond differently to food marketing techniques; food marketing has a greater effect on boy’s food choices and preferences compared to girls; and food advertisements on television contain more male characters^(15)^. The results of this scoping review suggest that research is needed to explore socially built stereotypes and how these may be leveraged in marketing to impact the food preferences of girls and boys. A noted limitation of the scoping review was the lack of evidence regarding digital marketing^(15)^. These results beg the question – are noted differences in both the amount and content of food marketing exposures contributing to gender-based health and diet disparities?

The gender dimension of health is a key analytical and explanatory variable in research. If gender is overlooked, our understanding of prevailing health issues will be incomplete and potentially biased, which may limit our ability to develop effective interventions and policies^(13)^. Compared with other commodities, such as alcohol and tobacco^(16)^ that have successfully utilised gender-based marketing strategies to draw in and maintain consumers, there is little research on the role gender plays in the design and impact of the digital marketing of unhealthy food.

Given that differences exist between boys and girls in obesity rates, dietary choices, and social media use, and that research indicates there are gender-based differences in responses to food marketing, it is critical to examine the digital marketing environment given the amount of time adolescents spend on social media and online. Such research is important to assess the messages that are shaping food attitudes among adolescent boys and girls, to provide insights as to whether gender differences in digital food marketing exposures play a role in overall dietary patterns and obesity prevalence, and to help inform digital marketing policies and interventions that are equitable and protective of youth. Recognising that targeted marketing techniques by gender may play a part in observed dietary differences and obesity prevalence among adolescents it is imperative that policies and interventions are gender-sensitive to curb gender-based targeting attempts from food companies and to ensure boys and girls are equally protected.

The objectives of this pilot study were to determine if adolescent boys and girls were exposed to different amounts of food marketing including the types of food categories, the healthfulness of food products and the marketing techniques used to promote foods on their favourite social media applications. It was hypothesised that marketing techniques used to promote food and beverages would differ by gender based on results from a recent scoping review.

## Methods

### Data source

This study is a secondary analysis of a cross-sectional study that was conducted by Potvin Kent *et al*., in 2018^(17)^, which sought to compare the frequency and healthfulness of food marketing exposures viewed by children and adolescents on their two preferred social media apps. Apps included Facebook, Instagram, Snapchat, Twitter and YouTube. The original study asked participants to fill out a self-administered questionnaire that requested sociodemographic characteristics, including gender. Participants were asked to identify their gender with the following response options: (1) boy; (2) girl; or (3) I do not identify as a boy or a girl. Those who chose option three were provided the option to self-identify. Participants were then asked to login to their two favourite social media apps for 5 min per application (10 min total) on the smartphone or tablet they usually use during their leisure time. Participants wore eye-tracking Tobii Pro Glasses while using a social media application, which recorded everything that participants viewed while browsing.

Research assistants then identified all food marketing exposures in the video footage. Food marketing exposures were defined as any content in which food or beverage brand logos or branded products were featured and included food advertisements (display and video ads as well as companies’ posts on social media shared by their corporate account or other users), celebrity-generated content (when food products or brand logos appear in content produced and shared by celebrities or well-known figures on social media that have a large following) and food marketing embedded in other web content (branded food products, logos or product placements seen in recipe videos, art and craft videos, media articles or programmes, videos of sport highlights, streamed television content and Snapchat subscription articles, among others). User-generated content, content uploaded and shared by a social media user that intentionally or unintentionally promoted a food brand or product, whether it was encouraged by food companies or not (e.g. Snapchat photo posted by a private account featuring a McDonald’s McFlurry) were excluded from the sample as this study was interested on the targeting nature of food companies and affiliates. Exposures were then classified by food categories including cold cereal, cakes, cookies, and ice cream, candy and chocolate, snacks, 100 % fruit juice, sugar-sweetened beverages (including regular soft drinks, sports drinks, fruit drinks, energy drinks and iced tea), hot beverages (tea or coffee), fast-food restaurants, non-fast-food restaurants, cheese, grocery store items, condiments, and other (items not categorised, such as beef broth). Food marketing exposures were also identified by food company and their healthfulness. A registered dietitian (EP) assessed each promoted food item displayed in an advertisement using the products nutritional data and the Pan American Health Organization Nutrient Profile Model (PAHO NPM)^(18)^. Nutritional data, including energy and nutrient content of promoted food items, were sourced from the following in order of priority: the Canadian company website, a products Nutrition Facts table, the American company website, or the Canadian Nutrient File. Information taken from these sources included serving size, total calories, total fat, saturated fat, trans-fat, sugar, carbohydrates, fibre, sodium and protein per serving size. The PAHO NPM classifies foods based on their level of processing and content in terms of ‘negative’ nutrients that are a public health concern (e.g. free sugars, sodium and fats)^(18)^. All food items, regardless of their level of processing, were coded as being excessive or not in total fat (if total fat accounted for ≥30 % of calories), saturated fat (≥10 % of calories), trans-fat (≥1 % of calories), sodium (mg: kcal ratio ≥ 1) and free sugars (≥10 % of calories)^(18)^. Marketing exposures were coded as either minimally processed or processed, or ultra-processed according to PAHO definitions^(18)^.

### Characteristics of participants and marketing exposures

The original study consisted of 101 participants of which, thirty-eight were children aged 7–11 years and sixty-three were adolescents aged 12–16 years^(17)^. This study included sixty-two adolescents (twenty-six boys and thirty-six girls). One adolescent was excluded because they did not identify as a boy or girl. Adolescents were selected as there is little research that has focused on this age group.

### Assessment of food marketing exposures for marketing techniques

A content analysis of each participant’s food and beverage marketing exposures was conducted to identify the presence of the marketing techniques described in Table 1.

**Table 1:** Marketing techniques descriptions and examples

Marketing technique	Description	Example
Presence of a character, superhero, cartoon, etc.	A cartoon, character, superhero, animal, creature, etc. (e.g. fictional or unspecified) is used to market the product.	Using batman to advertise string cheese.
Presence of an influencer	An influencer is considered a celebrity, popular vlogger and/or athlete that has the ability to influence others into buying a product.	Mr. Beast (an influencer) endorses Coca-Cola in his posts.
Presence of an influencer – Athlete	An athlete influencer is a subform of influencer marketing that uses an individual associated with sports to endorse or sell a product.	An athlete (e.g. Lebron James) is seen consuming or interacting with a food or beverage product (e.g. Lebron James shoots some hoops and then drinks a Gatorade).
Presence of an influencer – Celebrity	A celebrity is seen consuming or interacting with a food or beverage product.	Britney Spears is seen shooting a dance video and drinks a Pepsi afterwards.
Presence of an influencer – Vlogger	A vlogger (a person who regularly posts short videos to a vlog, like YouTube, is seen consuming or interacting with a food or beverage product).	PewDiePie is taping a streaming of himself playing online while eating M&M’s.
Presence of a branded character	A branded character is a fictional/cartoon character that is defined by a set of human attributes and characteristics to give the brand a unique personality.	Tony the Tiger, Pillsbury Doughboy, etc.
Presence of a licensed character	A license character involves licensing the rights from the owner of the cartoon character to place images on a product. For example, Using Spiderman on a luncheables package	Spiderman is used to market a product.
Presence of adolescents	Adolescents are present in the advertisement either in the form of a cartoon or real-life actors.	Adolescents are clearly visible interacting with the product.
Depiction of the product as a character	A food or beverage product in the advertisement is either animated or talking.	Talking cupcake or dancing pretzel.
Presence of adults	Adults are present in the advertisement either in the form of a cartoon or real-life actors. Adults can also be depicted as parents.	Adults are clearly present interacting with the product.
Gender of dominant product user is a woman or girl	The main person in the advertisement is a woman or girl.	The main person interacting with the product (e.g. holding it, consuming it, etc.) is a woman or girl.
Gender of dominant product user is man or boy	The main person in the advertisement is a man or boy.	The main person interacting with the product (e.g. holding it, consuming it, etc.) is a man or boy.
Presence of multiple genders	The advertisement showcases both genders being present in the advertisement either consuming or interacting with the product.	Both man/boy and woman/girl are present in the ad.
Promotion of product palatability	The advertisement highlights the products palatability through descriptors like taste, smell, texture, etc.	Starbucks coffee that describes its roast as bold, subtly sweet and smooth.
Promotion of product novelty	The advertisement highlights the products uniqueness by indicating it is trendy or a limited edition.	Limited edition Oreos.
Unconventional product	The advertisement highlights the products unconventional shape, colour, taste or combination of those characteristics.	Bundt cake full of cheese.
Appeals to convenience	The advertisement highlights the products convenience by indicating that it is easy to make or pack.	‘This trendy dessert is easier to make than you think’.
Appeals to affordability	The advertisement highlights the product’s affordability by indicating its product price or value for money.	‘$5 off any two chubby chicken burgers’.
Suggested users	The advertisement highlights suggested users for the product.	‘This product is great for your active teenager’.
Promotion of product quality	The advertisement highlights the product’s quality which can come in the form of its ingredients.	Tropicana – made with the freshest oranges.
Reference to energy	The advertisement specifically references how the product provides energy.	This product is great for energy or a quick pick-me-up.
Promotion of product desirability	The advertisement highlights the product’s desirability.	‘You can’t live without it!’
Appeals to hunger/thirst satisfaction	The advertisement highlights the product’s ability to satiate your hunger or quench your thirst.	Snickers commercial – ‘you’re not you when you’re hungry’.
Appeals to joy	The advertisement highlights the product in a way that appeals to fun, playfulness, happiness, humour or pleasure.	M&M commercials use humour to advertise their product.
Appeals to fantasy	The advertisement highlights the product in a way that appeals to fantasy, imagination or adventure.	Products that come alive and animations.
Appeals to social enhancement	The advertisement highlights the product’s ability to enhance making friends, peer acceptance or being social with others.	The Coca-Cola advertisement that features people giving friends Coke’s with their names on them.
Appeals to achievement	The advertisement highlights the product’s ability to help with achievement or accomplishment.	Mento’s that show problem-solving or achievement because of consuming the product. Grey Poupon showing status or achievement.
Appeals to coolness	The advertisement uses people that exhibit cool or hipness to market a product.	Using an individual leaning up against a wall holding a McDonald’s burger.
Appeals to athleticism	The advertisement highlights the products attributes to athleticism, referring to its ability to boost one’s strength, speed or sports performance or features individual(s) doing athletic activities with the product.	People snowboarding while drinking Mountain Dew.
Appeals to sex	The advertisement includes aspects of romance, sex or sexuality to market a product.	Paris Hilton wearing a bikini while eating a Carl’s Jr Burger.
Appeals to beauty	The advertisement includes aspects of beauty or attractiveness to market the product.	An influencer doing their make-up while consuming a drink or food product.
Appeals curiosity	The advertisement peeks a consumer’s interest by stimulating curiosity or asking questions that leave the viewer wondering.	Dorito’s ‘what happens when you open these snacks?’
Appeals to healthfulness	The advertised product appeals to healthfulness.	Displays a product that is healthy, or how the product will affect your health.
Women are specifically referenced	The advertisement specifically mentions either in writing or verbally that the product is more appealing to women.	‘This low-fat yogurt is great for any mom on the go’.
Men are specifically referenced	The advertisement specifically mentions either in writing or verbally that the product is more appealing to men.	‘Are you man enough for this steak?’
Nutrition-related claims	The advertisement specifically references or showcases a nutrient claim.	Fat-free, low Na, etc.
Health appeal claims	The advertisement specifically references or showcases a health appeal claim.	References or showcases a health appeal claim such as health symbols (e.g. hearts, checks), it references ‘natural/pure’, promotion of improved health outcomes (e.g. growth, weight loss), depicting ‘healthy’ people.
Association with healthy foods	The product is advertised to be composed of healthy foods such as whole grains, fruits and vegetables, etc.	Tropicana orange juice using only real Florida oranges.
Eating information	Eating information is made clearly visible in the advertisement.	Serving suggestions, eating guidelines, etc.
Featuring or reference to health professionals, health institutions, etc.	The advertisement specifically references health professional or institutions when marketing the product.	Doctor approved, etc.
Reference to unhealthy eating	The advertisement references aspects of unhealthy eating.	This product is high in calories or sugar or fat, etc.
Visual effects	Visual effects are used to market the product.	Animations that are fast cutting, slow motion or dynamic images are used.
Graphic imagery	Graphic imagery is used to enhance the display of the product.	Bright colours, eye-catching backgrounds, etc.
Direct reference to and/or use of word ‘teen(ager)’	The word or similar words to ‘teenager’ are used either verbally or visually to market the product.	‘Great for teens, your teen will love it’.
Use of slang	Slang terms are used throughout the advertisement.	‘Restaurants were too damn extra’, ‘treat yo self’.
Use of music	Music is used to market the product.	Songs, jingles, sound effects, etc.
Giveaways to be redeemed later	The product is advertised with an associated giveaway that can be redeemed later.	A contest, competition, coupon, special order items or product sample.
Price-related premiums	The product is advertised with a price-related premium or rebate.	A bonus offer or calls to action to encourage purchase.
Giveaways included with purchase	The product is advertised with a giveaway that is included with purchase.	Gifts, toys or collectibles (e.g. Happy Meals).
Other purchase incentives	The product is advertised with a purchase incentive.	Access to different areas of a website or the ability to move to a higher level of a game.
Presence of quizzes, surveys or polls	A quiz, survey or poll is included in the advertisement.	The viewer is provided two food options and is asked what product has more calories in the form of a poll.
Prompts to communicate with brand	The advertisement encourages further communication with the brand.	The viewer is encouraged to sign up for a newsletter, provide contact info, or an opportunity to communicate with a branded character.
Reference to entertainment	The advertisement ties in movies, TV, toys or pop culture references to market the product.	Danny Devito dressed up as an M&M for super bowl teaser.
Promotion of links	The advertisement includes links to the company or product website/apps.	Provides additional ways to access the product.
Promotion of links to other food/non-food websites	The advertisement promotes links to other foods or non-food websites within the advertisement.	Coca-Cola will link its other products within its coke ad.
Crossover with other brands	The advertisement displays or promotes other brands.	A tweet that says, ‘You be my dairy queen and I’ll be your burger king’.
Presence of a brand	The product logo or name and tagline is clearly displayed within the advertisement.	Tim Hortons – always fresh – is clearly displayed in the post.

These techniques were adapted from Mulligan *et al*. (2020)^(19)^. All food marketing exposures (*n* 139) and their marketing techniques were coded by a master coder. In this approach, one researcher (AA) served as the standard, while a second reviewer (MP) was used as a reliability coder. The reliability coder coded a subset of the total data set to establish inter-rater reliability with the master coder. In our study, the second reviewer (MP) coded a random 25 % sample^(20)^. Each reviewer identified the presence (or lack thereof) of marketing techniques for every food marketing exposure. The coding of marketing techniques between the two reviewers was compared, and inter-rater reliability was calculated using Cohen’s *κ*. The overall inter-rater reliability was found to be 0·71. According to McHugh’s (2012), 0·61–0·80 can be interpreted as having a substantial level of agreement^(21)^.

### Data analysis

Participants were classified as being exposed or not to elements of food marketing content (e.g. food category, PAHO classification, food marketing techniques, etc.). Descriptive statistics were then tabulated. Gender differences in the frequency and content of exposures were tested using a Mann–Whitney *U* test and Fisher’s exact tests, as appropriate, on 2 × 2 tables. Data were analysed using IBM SPSS v.27.0., 2020^(22)^.

## Results

Overall, there were sixty-two participants (*n* 36 girls, *n* 26 boys) in this study. Those who identified as a girl accounted for 58 % of participants, with 53 % being 12 or 13 years old. Boys accounted for 42 % of participants, with 38 % being 15 years of age. As shown in Table 2, 65 % of all participants were White, and over half (52 %) were from households whose annual income was $100 000 or more. Instagram (47 %) followed by Snapchat (44 %) were the preferred social media apps for girls, whereas boys most frequently used Instagram (35 %), followed by YouTube (31 %).

**Table 2:** Sociodemographic characteristics and social media use of all participants (*n* 62)

	Boys (*n* 26)		Girls (*n* 36)		Total (*n* 62)	
	*n*	%*	*n*	%*	*n*	%*
Age (years)						
12	6	23	10	28	16	26
13	5	19	9	25	14	23
14	4	15	8	22	12	19
15	10	38	7	19	17	27
16	1	4	2	6	3	5
Race						
White	15	58	25	69	40	65
Other	11	42	11	31	22	35
Income†						
Less than $50 k	2	8	5	14	7	11
$50 k to less than $100 k	7	27	2	6	9	15
$100 k to less than $150 k	5	19	8	22	13	21
Over $150 k	5	19	14	39	19	31
Refuse to answer	7	27	7	19	14	23
Social media app use						
Facebook	4	15	1	3	5	8
Instagram	9	35	17	47	26	42
Snapchat	3	12	16	44	19	31
Twitter	1	4	2	6	3	5
YouTube	8	31	2	6	10	16

* Note that the sum of %’s do not add up to 100 due to rounding.

† Income before taxes and deductions.

### Exposures to food marketing

Of the sixty-two participants in this study, forty-seven (76 %) were exposed to an instance of food and beverage marketing. The number of exposures viewed by participants did not differ significantly between boys and girls (median = 2 for boys and girls, respectively, Mann–Whitney *U* = 237, *P* = 0·51) (data not shown). As shown in Table 3, the source of food marketing exposure viewed most by participants was food advertisements (50 %) followed by food marketing embedded in other web content (40 %). More boys viewed food advertising (54 % *v*. 47 %) and celebrity-generated content (15 % *v*. 6 %) compared to girls, whereas a greater share of girls viewed instances of food marketing embedded in other web content (47 % *v*. 31 %) compared to boys. Gender differences in exposure to food marketing sources were not statistically significant.

**Table 3:** Participant’s exposure source to food marketing in 10 min of social media use, by gender

Type of food marketing	Boys (*n* 26)		Girls (*n* 36)		Total (*n* 62)		Fisher’s exact test
	*n*	%	*n*	%	*n*	%	*P*-value
Food advertisements	14	54	17	47	31	50	0·8
Celebrity-generated content	4	15	2	6	6	10	0·23
Food marketing embedded in other web content	8	31	17	47	25	40	0·29

### Food categories

As shown in Table 4, the food categories most viewed by participants were fast foods (50 % of participants), cakes, cookies, and ice cream (19 %), sugar-sweetened beverages (19 %) and candy and chocolate (16 %). Half (50 %) of all boys viewed instances of marketing promoting fast foods, followed by sugar-sweetened beverages (27 %), non-fast-food restaurants (12 %) and condiments (12 %), while half of girls (50 %) viewed instances of marketing promoting fast foods, followed by cakes, cookies, and ice cream (28 %), candy and chocolate (22 %), and snacks (17 %). Gender differences in exposure to food categories were not statistically significant.

**Table 4:** Participants exposures to food categories in 10 min of social media use, by gender

Food categories	Boys (*n* 26)		Girls (*n* 36)		Total (*n* 62)		Fisher’s exact test
	*n*	%*	*n*	%*	*n*	%*	*P*-value
Fast-food restaurants	13	50	18	50	31	50	1
Cakes, cookies and ice cream	2	8	10	28	12	19	0·06
Sugar-sweetened beverages	7	27	5	14	12	19	0·5
Candy and chocolate	2	8	8	22	10	16	0·17
Snacks	1	4	6	17	7	11	0·22
Non fast-food restaurants	3	12	4	11	7	11	1
Condiments	3	12	2	6	5	8	0·64
100 % fruit juice	2	8	1	3	3	5	0·57
Grocery store items	2	8	0	0	2	3	–
Cold cereal	1	4	0	0	1	2	–
Hot beverages	1	4	0	0	1	2	–
Cheese	1	4	0	0	1	2	–
Other†	4	15	4	11	8	13	0·71

* It is possible for more than one food item to be present in a single ad; therefore, percentages will be higher than 100.

† Items not categorised in the above, such as beef broth.

### Healthfulness

**Fig. 1:**
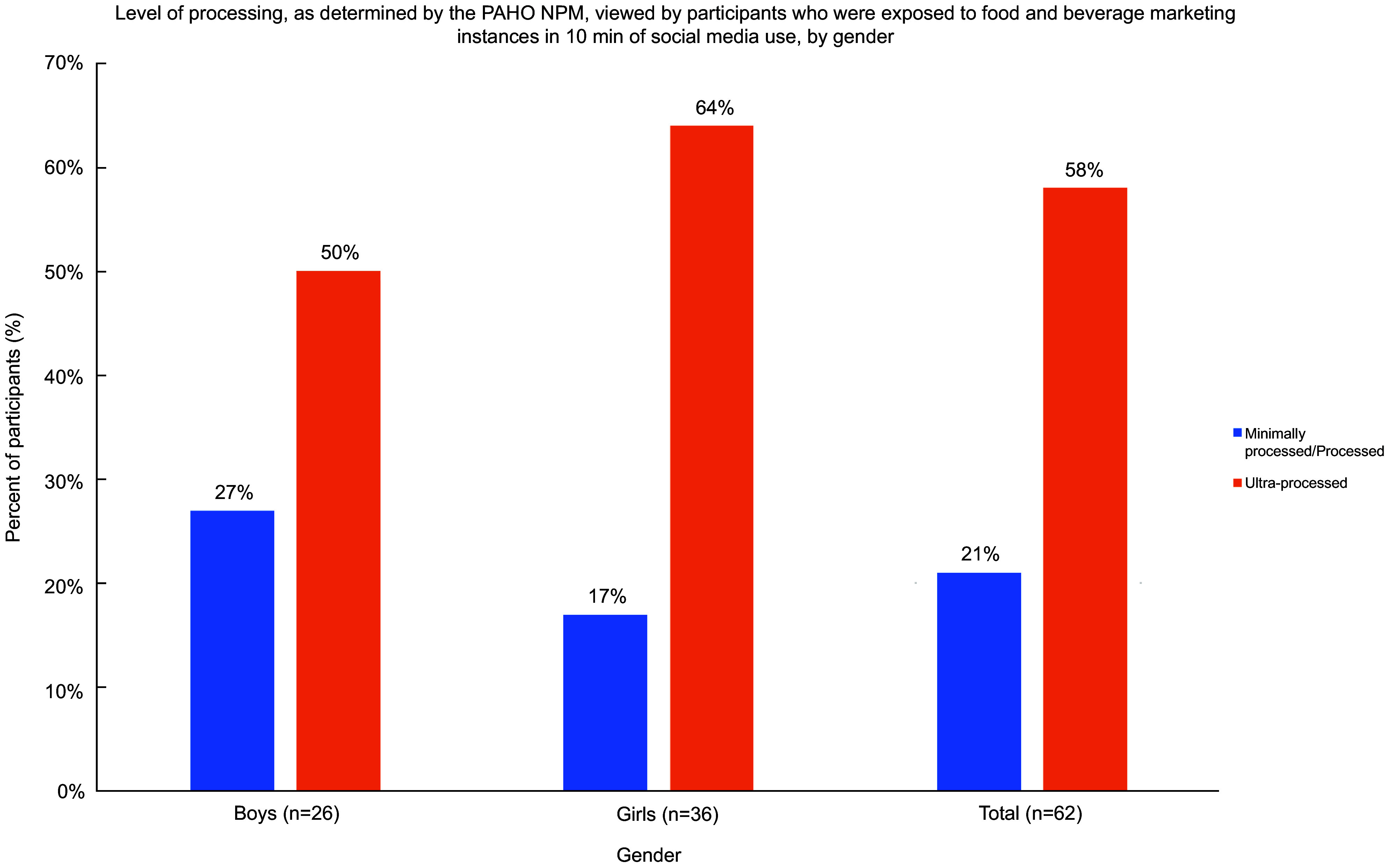
Percentage of participants exposed to products classified as minimally processed/processed and ultra-processed, as determined by the PAHO NPM, in instances of food marketing viewed during 10 min of social media use, by gender

As shown in Fig. 1, 64 % of girls viewed instances of food marketing exposures containing ultra-processed food items compared with 50 % of boys, whereas 27 % of boys viewed instances of minimally processed/processed items compared with 17 % of girls. Gender differences in exposure to food healthfulness were not statistically significant.

Over half of participants (63 %) viewed products that contained excessive amounts of at least one nutrient. As shown in Table 5, 53 % of participants viewed instances of marketing containing total fat, followed by saturated fat (52 %) and free sugars (44 %). More girls viewed products that were excessive in total fat compared to boys (67 % *v*. 35 %, *P* = 0·02). Overall, girls were more likely to view marketing instances containing greater amounts of each nutrient compared to boys. With the exception of total fat, none were found to be statistically significant.

**Table 5:** Participants exposure to healthfulness of products, as determined by the PAHO NPM, viewed by participants in 10 min of social media use, by gender

PAHO nutrient profiling	Boys (*n* 26)		Girls (*n* 36)		Total (*n* 62)		Fisher’s exact test
	*n*	%	*n*	%	*n*	%	*P*-value
Excessive in total fat	9	35	24	67	33	53	0·02
Excessive in saturated fat	11	42	21	58	32	52	0·3
Excessive in trans-fat	5	19	8	22	13	21	1
Excessive in Na	10	38	16	44	26	42	0·8
Excessive in free sugars	11	42	16	44	27	44	1
Excessive in at least one nutrient	14	54	25	69	39	63	0·29

### Marketing techniques

As demonstrated in Table 6, overall, the presence of a brand (68 % of participants), graphic imagery (56 %) and appeals to joy (53 %) were the most viewed marketing techniques by all participants. Boys were significantly more likely to view instances of food marketing where the gender of the dominant product user was a man or boy (50 % *v*. 22 %, *P* = 0·03), exposures appealed to achievement (42 % *v*. 17 %, *P* = 0·04) or athleticism (35 % *v*. 11 %, *P* = 0·03), and exposures featured an influencer (42 % *v*. 14 %, *P* = 0·02). Girls were significantly more likely to view instances of food marketing featuring quizzes, surveys or polls (25 % *v*. 0 %, *P* = 0·01).

**Table 6:** *Participant exposures to marketing techniques in 10 min of social media use, by gender

Marketing techniques	Boys (*n* 26)		Girls (*n* 36)		Total (*n* 62)		Fisher’s exact test
	*n*	%†	*n*	%†	*n*	%†	*P*-value
Presence of a brand	18	69	24	67	42	68	1
Graphic imagery	14	54	21	58	35	56	0·8
Appeals to joy	14	54	19	53	33	53	1
Appeals to hunger/thirst satisfaction	10	38	22	61	32	52	0·12
Presence of adults	14	54	15	42	29	47	0·44
Appeals to coolness	12	46	14	39	26	42	0·61
Visual effects	8	31	18	50	26	42	0·19
Appeals to social enhancement	12	46	13	36	25	40	0·45
Gender of dominant product user is man or boy	13	50	8	22	21	34	0·03
Promotion of product novelty	7	27	10	28	17	27	1
Promotion of product desirability	5	19	12	33	17	27	0·26
Appeals to achievement	11	42	6	17	17	27	0·04
Appeals to curiosity	7	27	10	28	17	27	1
Crossover with other brands	6	23	11	31	17	27	0·58
Presence of an influencer	11	42	5	14	16	26	0·02
Gender of dominant product user is a woman or girl	4	15	12	33	16	26	0·15
Appeals to healthfulness	6	23	10	28	16	26	0·77
Reference to unhealthy eating	6	23	9	25	15	24	1
Presence of a character, superhero and cartoon	3	12	10	28	13	21	0·21
Unconventional product	3	12	10	28	13	21	0·21
Appeals to athleticism	9	35	4	11	13	21	0·03
Promotion of product palatability	3	12	9	25	12	19	0·21
Use of slang	3	12	8	22	11	18	0·33
Promotion of links	6	23	5	14	11	18	0·5
Association with healthy foods	4	15	6	17	10	16	1
Presence of an influencer – Celebrity	6	23	3	8	9	15	0·15
Presence of quizzes, surveys or polls	0	0	9	25	9	15	0·01
Presence of multiple genders	3	12	5	14	8	13	1
Appeals to sex	3	12	5	14	8	13	1
Promotion of product quality	4	15	3	8	7	11	0·44
Reference to entertainment	3	12	4	11	7	11	1
Presence of an influencer – Athlete	5	19	1	3	6	10	0·07
Appeals to affordability	3	12	3	8	6	10	0·69
Appeals to fantasy	1	4	5	14	6	10	0·39
Depiction of the product as a character	1	4	4	11	5	8	0·39
Appeals to convenience	1	4	4	11	5	8	0·39
Nutrition-related claims	2	8	2	6	4	6	1
Use of music	2	8	2	6	4	6	1
Giveaways to be redeemed later	3	12	1	3	4	6	0·3
Presence of an influencer – Vlogger	2	8	1	3	3	5	0·57
Presence of adolescents	1	4	2	6	3	5	1
Suggested users	1	4	2	6	3	5	1
Appeals to beauty	1	4	2	6	3	5	1
Health appeal claims	1	4	2	6	3	5	1
Eating information	0	0	2	6	2	3	–
Other purchase incentives	0	0	2	6	2	3	–
Promotion of links to other food/non-food websites	0	0	2	6	2	3	–
Presence of a licensed character	1	4	0	0	1	2	–
Reference to energy	1	4	0	0	1	2	–
Price-related premiums	1	4	0	0	1	2	–
Prompts to communicate with brand	1	4	0	0	1	2	–

* Any technique not listed above was not viewed within an exposure by participants.

† It is possible for more than one technique to be present in a single ad; therefore, percentages will be higher than 100.

## Discussion

This study provides preliminary insights into the content adolescent boys and girls viewed on their two favourite social media apps according to source of marketing exposure, the healthfulness and food category of viewed food products, and marketing techniques. As hypothesised, marketing techniques used to promote food and beverages differed by gender. Our results also suggest that gender differences may exist with respect to healthfulness of promoted foods.

### Differences in food categories

Fast foods were the most frequently viewed food category in our study, with half of participants having viewed a food marketing instance containing a fast-food item. This is to be expected, as fast foods are regularly one of the most marketed food products to adolescents, highlighting a universal pattern^(10,11,23,24)^. The ubiquity of fast-food marketing is problematic as there is a demonstrated relationship between marketing exposures, food choice and increased consumption of unhealthy foods after social media use^(25)^. The volume of online fast-food marketing is concerning as excessive consumption of these items are associated with an increased risk of obesity, CVD and diabetes^(26)^.

### Differences in healthfullness

The results of our content analysis also revealed that 64 % of girls viewed marketing exposures that contained ultra-processed foods. Like this study, a small, exploratory study examining the food messages Flemish adolescents were exposed to on social media found that ultra-processed foods, such as fast foods, made up 67 % of the messages they viewed^(27)^. Food items that are ultra-processed have low nutritional quality, are highly palatable and typicaly require little culinary preparation making them convenient and appealing to adolescents^(26)^. In 2015, nearly 50 % of daily caloric intake for Canadian females aged 13–18 years and 53 % of daily caloric intake for males aged 13–18 years were composed of ultra-processed foods^(28)^.

Marketing is powerfully influential in not only reflecting norms and gender perceptions, but in shaping them^(29)^. Social and cultural norms are a key component in the formation of food preferences^(30)^ and as youth seek independence from family and conform to the norms of their surroundings, including what is viewed on social media, they are at risk of adopting unfavourable health behaviours, such as the excessive consumption of ultra-processed foods. The frequency at which food marketing is displayed can create normative views regarding diet^(27)^. High frequencies of marketing containing ultra-processed foods can easily influence attitudes about diet and eating behaviours. Food and beverage companies may be using marketing techniques that are more appealing to girls and women in attempts to increase consumption of ultra-processed foods to a group that is traditionally health conscious^(31)^. This type of targeting may be an effort to rewrite existing social norms where girls are more concerned with dieting and weight loss by exposing them to greater amounts of unhealthy food in attempts to increase consumption.

The emerging picture of digital marketing is one of an environment that heavily promotes and normalises the overconsumption of ultra-processed food^(27)^. For this reason, it is imperative that researchers explore the methods that companies use to target consumers based on gender to develop specific food marketing restrictions. Although the diets of women and girls have historically been healthier^(32)^ as they are generally more health conscious than boys, the propagation of ultra-processed food products through social media could be shaping a new reality. If continued unabated, digital marketers may rewrite the norms of dietary habits for young girls, resulting in an increase of obesity and other chronic disease rates.

### Gender differences in exposure to various marketing techniques

One of the most significant findings of this study was that exposures to marketing techniques, as hypothesised, differed by gender. An interesting result was that boys viewed food marketing instances where the dominant product user was a man or boy. These findings are congruent from Ogle *et al.*’s experimental study where they found children preferred food products where the packaging depicted a licensed cartoon character that represented their gender^(33)^. Using gender and gender stereotypes creates relatability, which helps establish a sense of connection to the product, translating into increased awareness and sales^(34)^. A study conducted by Higgins *et al.* (2018) concluded that advertising campaigns that used age and gender data were able to achieve statistically significant boosts in engagement by using gender-specific marketing strategies^(35)^. This suggests that the use of gender in marketing is deliberate and can be used to entice a viewer based on their gender and is evidence of individualised, gender-specific, curated marketing. Further, evidence from alcohol and tobacco studies suggests that people can be influenced by marketing that models qualities, such as gender, that consumers find relatable^(36,37)^. When an adolescent positively identifies with the person promoting a food product, an individual’s gender in this case, their food choices and consumption habits favour the product regardless of healthfulness^(11)^. Taking a page from tobacco and alcohol, food marketers, therefore, could be leveraging existing sex and/or gender differences to impact behaviour, resulting in the reinforcement of sex-/gender-based differences and deepening sex-/gender-based preferences for particular foods^(32)^.

Our study also found that boys were significantly more likely to view instances of food marketing that featured appeals to achievement and athleticism and include the presence of an influencer. Achievement, athleticism and the utilisation of masculinity were themes uncovered in a study that explored how sugar-sweetened beverages were marketed to Australian youth through branded Facebook pages^(39)^. Showcasing achievement and athleticism in food and beverage marketing strategically aligns a product within sociocultural values and practices found important to the male demographic^(39)^. In doing so, food and beverage companies portray their products as having a regular place within the consumers’ everyday life. This type of manipulation is concerning given the impressionable nature of adolescents^(11)^ and the associations that can be developed between male stereotypes and unhealthy food.

Influencers, including athletes and celebrities, are often used to market products^(7)^. A study that investigated pre-adolescent children’s responses to child-oriented front-of-pack food promotions found that sports celebrity endorsements of unhealthy food products persuaded participants preferences for those items over healthy food or non-food options^(40)^. Boys favoured packages that focused on athletics, which traditionally demonstrates stereotypical masculine characteristics like athleticism and strength^(38,40)^. Using an influencer, like a famous athlete, to market food and beverages also creates the added appeal of achievement, which can result in the viewer forming an association between consuming the product and being successful. Using influencers like sports figures to market unhealthy food and beverages can create a ‘health halo’, deceiving adolescents into false impressions of these items being healthy^(41)^. These findings are troubling as celebrities and influencers were found to promote unhealthy foods significantly more often than healthy foods^(11)^.

Conversely, a significantly greater proportion of girls viewed food marketing exposures that used polls, surveys or questionnaires. This could be due to the platforms used by this study’s participants, as polls, surveys and questionnaires are often found on Snapchat Discover^(42)^. Research supports that there is a difference in how females and males respond to online surveys^(43)^. Gender can shape behaviour with females being more likely to engage in online activity characterised by communication and exchanging of information (surveys), whereas males are more likely to engage in online activity characterised by seeking information^(44)^. Companies may use the tactic of surveys, polls and questionnaires as hooks to entice women to interact with their products. Young women interacting with food and beverage company surveys, polls and questionnaires may be less aware of the promotional intent of the marketing as it is being veiled as interactive content^(44)^. Further, adolescents who are more engaged with online content are more likely to share and create similar content of their own, which can reinforce and/or establish food and diet social norms^(7)^.

### Targeted marketing

Half of all participants in this study were exposed to food advertisements. Digital marketing creates unique challenges in restricting youth’s exposure to unhealthy foods. Unlike television, each social media user views a different set of food marketing instances depending on their food preferences and previous interactions with a brand or other content^(45)^. Research conducted by the *UConn Rudd Center for Food Policy and Obesity* in 2019 documented the increasing amount of unhealthy food advertising that targets Hispanic and Black youths^(46)^. The report highlighted that multicultural food marketing, marketing that is designed to appeal to individuals of different racial and/or ethnic groups, continues to disproportionately target youth of colour and contributes to health disparities affecting these populations, the impetus of which should be applied to gender. Multicultural targeting raises public health concerns and perpetuates negative diet-related health disparities affecting ethnic populations^(45,46)^. Conclusions drawn from multicultural food marketing and results from our research highlight the importance of evaluating how companies and affiliates are targeting specific demographics, potentially perpetuating health disparities.

The marketing of other commodities, such as tobacco and alcohol, have regularly and successfully used gender-targeted marketing. Alcohol and tobacco companies have long been attracting new consumers by developing marketing strategies that exploit gender norms and stereotypes^(36)^. For example, the tobacco industry put in a concerted effort towards increasing the female consumer base by creating advertisements that use beauty to draw women’s attention^(36)^. Slim cigarettes advertised as feminine are one example of this approach. Alcohol advertisements have used targeted segmentation, which is a strategy used for isolating gender differences to make advertisements more appealing to an intended audience^(37)^. Examples of segmentation techniques include using imagery and emotional connections, which have been shown to effect men and women differently^(37)^. Being cognizant of targeting strategies that utilise gender stereotypes is important for informing polices that are protective of the targeted audience.

Food products become gendered as a result of marketing strategies that are tailored towards a specific gender. Branding and promotional techniques are utilised in a way where a product essentially becomes ‘food for boys’ or ‘food for girls’. Overall, a greater share of boys in this study viewed instances of targeted marketing techniques, compared with girls. An explanation for these observed differences might be the proportion of males used in marketing, and that marketing tends to focus on boys because they are more susceptible to food advertisements^(47)^.

Our study suggests that gender differences may be playing a role in the design of marketing strategies, which could be leading to the perpetuation of unhealthy eating behaviours, creating gender disparities in food choices and overall health. Gender bias may exist, underscoring the need for solutions and future research that emphasise the role gender plays in food marketing. Additional research that directly tests promotional strategies designed to elicit gendered engagement would allow for more conclusive results and would eliminate attentional or consumption bias.

### Strengths and limitations

To our knowledge, this is the first study that examines the digital marketing techniques adolescents viewed, according to their gender. Given the exploratory nature of this research, there are several limitations. Participants’ exposure to food marketing on their favourite social media sites were only captured over a 10-min period. The short duration of the viewing time is not representative of the average viewing time adolescents spend on social media applications. Further, newer social media apps like TikTok and Twitch were not captured in this study but have been shown to impact attitudes and behaviours in relation to food and beverage marketing^(48)^. Additionally, this study only included a small convenience sample of participants, where 65 % of participants were White, and over half were from households whose annual income was $100 000 or more. Participants were also only recruited from Ottawa, and as a result, findings may not be generalisable to the Canadian population. This study did not consider confounding variables, and as such observed differences between gender groups could be attributable to other factors such as differences in age. Lastly, the full scope of marketing exposures including both food and non-food marketing instances was not captured. Insights into the proportion of digital food and beverage content adolescents are exposed to compared with non-food content would provide a more fulsome picture of the online food environment.

Despite this, the results of our research provide preliminary insights into gender targeting being used to digitally market unhealthy food and beverage products to adolescents. Awareness of these differences is important for understanding food choices and subsequent health differences between boys and girls. The results of this study can provide insights into programmes and policies that protect adolescents of different genders from targeted marketing techniques. Determining how to better identify gender-specific marketing appeals will allow for the monitoring of message content that can inform regulatory policies on food marketing to adolescents. Given the paucity of research that explores digital food and beverage gender-based marketing techniques directed at adolescents, a broad range of research is required. Future research would benefit from using a larger sample size and investigating how adolescents, based on gender, perceive the food marketing exposures they are engaging with over social media applications. This exploration would provide valuable insights into how adolescents personally interact with food and beverage companies, helping draft regulations that are informed by those most impacted.

## Conclusion

To address the growing obesity epidemic, the World Health Organisation (WHO) recommends restricting unhealthy food advertising to youth, compelling states to implement policies and regulations that limit child and adolescent’s exposure to the marketing of unhealthy, nutrient-poor foods^(49)^. Stringent regulation is needed to address the persuasive tactics used by food and beverage companies that target adolescents and children. Just as marketing is most effective when it targets specific groups or individuals, so too is the creation of public health policies and regulations. Gender is a determinant of health and therefore must be considered in digital food and beverage marketing research and future policies that address obesogenic environments.

## Supplementary material

For supplementary material accompanying this paper visit https://doi.org/10.1017/S1368980022002312. click here to view supplementary material
