# Supplementary material for: Adolescent exposure to food and beverage marketing on social media by gender: a pilot study
Source: Public Health Nutr. 2022 Nov 2;26(1):33–45. doi: 10.1017/S1368980022002312 (PMC11077454; doi:10.1017/S1368980022002312)
Supplement: Supplementary file 1 [file S1368980022002312sup001.docx]

**Supplemental Table**

*Food companies*

As shown in Supplemental Table 1 , McDonald’s (24%), PepsiCo (21%), and Mars (21%), were the most viewed food companies by all participants. Boys viewed McDonald’s (23%), Pepsico (23%), and Restaurant Brands International (19%), while girls viewed instances of food marketing containing McDonald’s (25%), PepsiCo (19%), and Mars, Mondelez, and Starbucks (19%).

Supplemental Table 1. Participant’s exposure to food companies in 10 minutes of social media use, by gender

|  | **Boys** | **Girls** | **Total** | **Fisher's exact test** |
| --- | --- | --- | --- | --- |
|  | **(n=26)** | **(n=36)** | **(n=62)** | **p value** |
| **Food company** | **n(%)** | **n(%)** | **n(%)** |  |
| McDonalds | 6(23) | 9(25) | 15(24) | 1 |
| PepsiCo | 6(23) | 7(19) | 13(21) | 0.76 |
| Mars | 2(8) | 7(19) | 9(15) | 0.28 |
| Mondelez | 1(4) | 7(19) | 8(13) | 0.12 |
| Starbucks | 2(8) | 6(17) | 8(13) | 0.45 |
| Restaurant Brands International | 5(19) | 1(3) | 6(10) | 0.07 |
| Kraft | 1(4) | 3(8) | 4(6) | 0.63 |
| Maple Leaf Foods | 1(4) | 3(8) | 4(6) | 0.63 |
| Wendy's | 1(4) | 3(8) | 4(6) | 0.63 |
| Doctor's Association | 2(8) | 1(3) | 3(5) | 0.57 |
| Other companies | 12(46) | 16(44) | 28(45) | 1 |
